# Supplementary material for: Social behaviour and vocalizations of the tent-roosting Honduran white bat
Source: PLoS One. 2021 Aug 11;16(8):e0248452. doi: 10.1371/journal.pone.0248452 (PMC8357122; doi:10.1371/journal.pone.0248452)
Supplement: S1 File — (PDF) [file pone.0248452.s004.pdf]

Supporting information for

Social behaviour and vocalizations of the tent-roosting Honduran White Bat

Ahana Aurora Fernandez[1*], Christian Schmidt[2], Stefanie Schmidt[2], Bernal Rodríguez-Herrera[3+], Mirjam Knörnschild[1,4,5+*]

[1] Museum für Naturkunde - Leibniz Institute for Evolution and Biodiversity Science, Berlin, Germany

[2] Institute of Conservation Genomics, University of Ulm, Germany

[3] Escuela de Biología, Centro de Investigación en Biodiversidad y Ecología Tropical, Universidad de Costa Rica, San José, Costa Rica

[4] Smithsonian Tropical Research Institute, Balboa, Panama

[5] Animal Behavior Lab, Freie Universität Berlin, Germany

[+] Joint senior authors

*Corresponding authors: ahana.fernandez@mfn.berlin, mirjam.knoernschild@mfn.berlin

Content

Video S1. Male fur chewing.

Video S2. Pup play behaviour.

Dataset S1: 22-hour observation period of group 1.

Fig S1. Spectrograms of social call types SC9 and SC10.

Table S1. Assessment of model fit of the discriminant function analysis including acoustic measurements of the loudest harmonic (DFA1).

Table S2. Correlations between acoustic parameters and standardised canonical discriminant functions (DF1-DF4 of DFA1).

Table S3. Classification success [%] of the cross-validated discriminant function analysis for eight social call types including measurements of F0 (DFA2)

Table S4. Assessment of model fit of the discriminant function analysis including acoustic measurements of F0 (DFA2).

Table S5. Correlations between acoustic parameters and standardised canonical discriminant functions (DF1-DF4 of DFA2).

Video S1. Male fur chewing.

This video captures the pup and an adult male together in the roost at night (the other four individuals of this social group are absent). Directly after landing in the tent, the male briefly smells the pup and directs himself behind the pup. Immediately, he starts biting/chewing the pup´s fur. The biting is accompanied by wing trembling. The pup seems irritated, trying to move around. At some point, it attempts to stretch its wing. After a few seconds, the male starts cleaning himself. At the end of the video the pup turns around and a wet part can be spotted on its back.

Video S2. Pup play behaviour.

This video captures the pup alone in the roost at night. The detailed description of the pup´s behaviour can be found in the results section of the study.

Dataset S1: 22-hour observation period of group 1.

This Excel sheet includes information on behavioural states and events observed during the 22-hour observation period of the four individuals of group 1.

Fig S1.

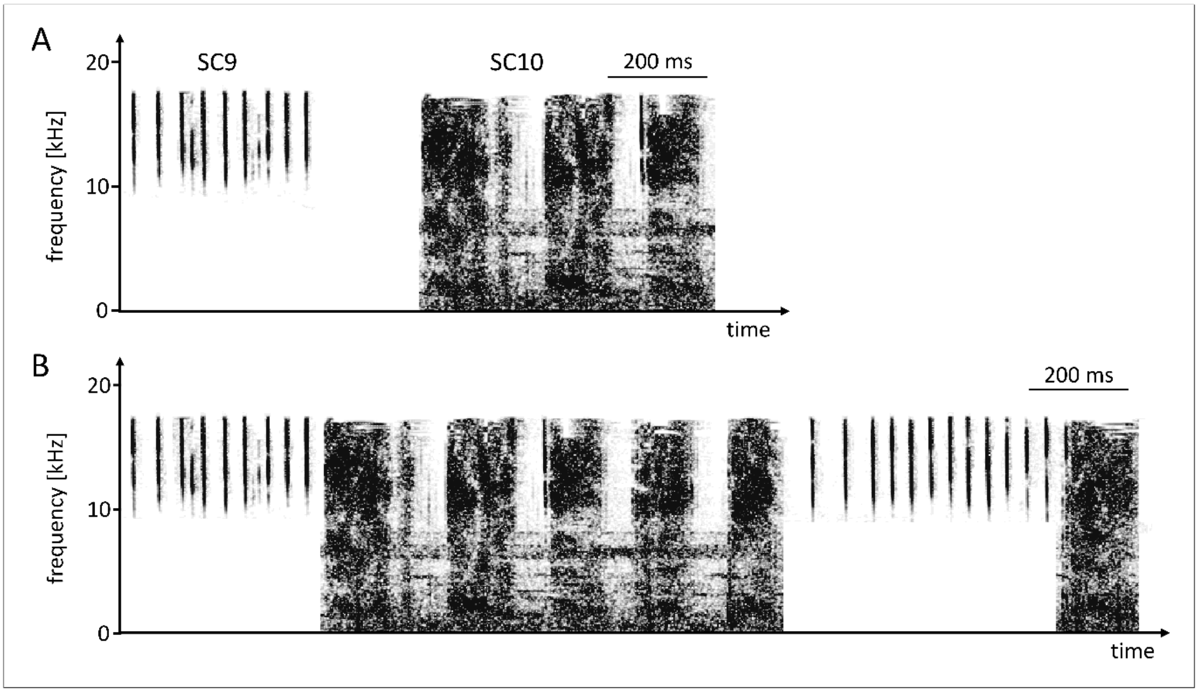

Fig S1. Spectrograms of social call types SC9 and SC10.

Spectrograms depicting the social call types SC9 and SC10 of *E. alba* **(A)**. Both social call types were emitted by an adult male in response to a mosquito sting on a hairless spot on the back of the bat. The vocalizations were accompanied by agonistic behaviour, including wing flapping and hitting conspecifics with folded wings. Both social call types were emitted in series and both were concatenated to a vocal sequence **(B)**. Both social call types were recorded only with the camcorder´s built-in microphone and not with the high-quality ultrasonic recording setup. This is the reason why the recordings are clipped at 20 kHz. Spectrograms were created using a 512-point FFT and a Hamming window with 75% overlap.

Additional information about discriminant function analysis including acoustic measurements of the loudest harmonic (DFA1).

Table S1. Assessment of model fit of the discriminant function analysis.

**Function**	**Eigenvalue**	**% of variance**	**Test of function**	**Wilkins λ**	**X^2**	**df**	***p***
1	15.184	69.6	1 to 4	0.003	631.563	28	< 0.0001
2	4.944	22.7	2 to 4	0.049	328.103	18	< 0.0001
3	0.914	4.2	3 to 4	0.293	133.825	10	< 0.0001
4	0.783	3.6	4	0.561	63.046	4	< 0.0001

Table S2. Correlations between acoustic parameters and standardised canonical discriminant functions (DF1-DF4).

**Acoustic parameter**	**DF1**	**DF2**	**DF3**	**DF4**
Duration (ms)	-0.120	0.550	0.788*	0.249
Peak frequency start (kHz)	0.731*	-0.459	0.309	0.400
Peak frequency centre (kHz)	0.894*	0.153	0.207	-0.367
Peak frequency end (kHz)	0.684*	0.646	-0.247	0.231

*Highest absolute correlation between every parameter and a DFA.

Additional discriminant function analysis including acoustic measurements of F0 (DFA2)

In addition to the discriminant function analysis (DFA) results reported in the study, we calculated a second DFA including acoustic measurements of the fundamental frequency of each social call instead of its most prominent harmonic.

The visual classification of eight social call types was confirmed by the classification success of the cross-validated DFA (92.2% of all call types were classified correctly, Table S3).

Table S3. Classification success [%] of the cross-validated discriminant function analysis for eight social call types including measurements of F0.

Social calls	SC1	SC2	SC3	SC4	SC5	SC6	SC7	SC8	No. of calls
**SC1**	100.0 (20)	0.0	0.0	0.0	0.0	0.0	0.0	0.0	20
**SC2**	0.0	100.0 (8)	0.0	0.0	0.0	0.0	0.0	0.0	8
**SC3**	0.0	0.0	91.7 (11)	0.0	0.0	0.0	8.3 (1)	0.0	12
**SC4**	0.0	0.0	0.0	100 (15)	0.0	0.0	0.0	0.0	15
**SC5**	0.0	0.0	0.0	0.0	60.0 (3)	0.0	0.0	40.0 (2)	5
**SC6**	0.0	2.4 (1)	0.0	0.0	0.0	97.6 (40)	0.0	0.0	41
**SC7**	0.0	0.0	0.0	33.3 (3)	0.0	0.0	66.7 (6)	0.0	9
**SC8**	0.0	0.0	0.0	0.0	16.7 (1)	0.0	16.7 (1)	66.7 (4)	6

Classification table showing how many call types were correctly classified to social call types. The number in each box indicates the correct classification of each correct call type (row) assigned to each predicted call type (column), i.e. each row sums up to 100 %. Numbers in brackets depict the number of calls (total call number: N=116). The total number of measured calls per type is indicated to the right of the matrix.

The acoustic parameters that contributed most to the distinction of social call types were peak frequency in the middle of the call and peak frequency at the start of the call, whereas end peak frequency and duration only played a minor role, equal to the DFA reported in the results section of the manuscript (Table S5).

Table S4. Assessment of model fit of the discriminant function analysis including acoustic measurements of F0.

Function	Eigenvalue	% of variance	Test of function	Wilkins λ	X^2	df	*p*
**1**	18.727	72.5	1 to 4	0.002	675.131	28	< 0.0001
**2**	5.063	19.6	2 to 4	0.40	350.092	18	< 0.0001
**3**	1.122	4.3	3 to 4	0.244	153.660	10	< 0.0001
**4**	0.930	3.6	4	0.518	71.646	4	< 0.0001

Table S5. Correlations between acoustic parameters and standardised canonical discriminant functions (DF1-DF4).

Acoustic parameter	DF1	DF2	DF3	DF4
Duration (ms)	-0.271	0.116	0.468	0.833*
Peak frequency start (kHz)	0.911*	-0.262	0.086	0.307
Peak frequency centre (kHz)	0.801*	0.336	-0.286	0.406
Peak frequency end (kHz)	0.540	0.620*	0.557	-0.122

*Highest absolute correlation between every parameter and a DFA.
