# Supplementary figures and images for: Social behaviour and vocalizations of the tent-roosting Honduran white bat
Source: PLoS One. 2021 Aug 11;16(8):e0248452. doi: 10.1371/journal.pone.0248452 (PMC8357122; doi:10.1371/journal.pone.0248452)

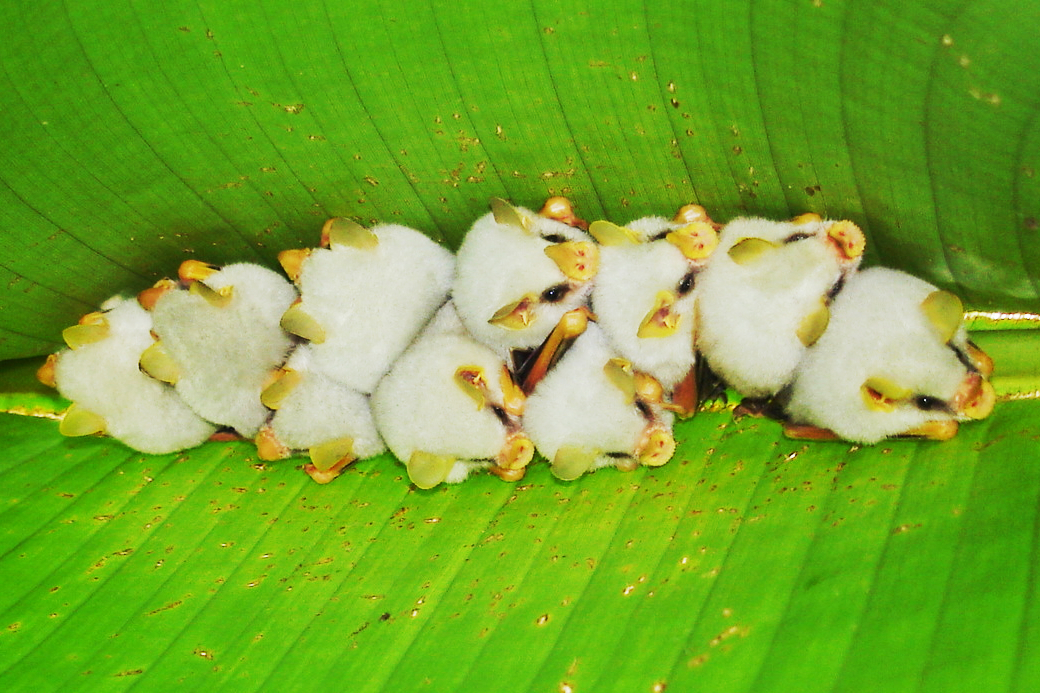

Supplement: S2 File — (JPEG) [file pone.0248452.s005.jpeg]
